# A PARK2 polymorphism associated with delayed neuropsychological sequelae after carbon monoxide poisoning

**DOI:** 10.1186/1471-2350-14-99

**Published:** 2013-09-25

**Authors:** Fei Liang, Wenqiang Li, Ping Zhang, Yanxia Zhang, Jiapeng Gu, Xiahong Wang, Hongxing Zhang, Renjun Gu

**Affiliations:** 1 Department of Neurology, the Second Affiliated Hospital of Xinxiang Medical University, Xinxiang 453002, China; 2 Henan Key Lab of Biological Psychiatry, Xinxiang Medical University, No.388, Jianshe Middle Road, Xinxiang 453002, China; 3 Health team of the 93123 unit, the Chinese People’s Liberation Army, Dalian, China; 4 Xinxiang Central Hospital, Xinxiang, China

**Keywords:** Delayed neuropsychological sequelae, Acute carbon monoxide poisoning, PARK2, SNP

## Abstract

**Background:**

Delayed neuropsychological sequelae (DNS) are the most severe and clinically intractable complications following acute carbon monoxide (CO) poisoning. Symptoms of DNS often resemble those of Parkinson’s disease (PD), suggesting shared neurological deficits. Furthermore, Parkinson protein 2 (PARK2) mutations are associated with PD and other neurodegenerative diseases. The association signal was detected between PARK2 and DNS after acute CO poisoning in our DNA pooling base genome-wide association study.

**Methods:**

Two PARK2 single nucleotide polymorphisms (SNPs), rs1784594 (C/T allele) and rs1893895 (G/A allele), selected from DNA pooling base genome-wide association study, were genotyped by in 514 CO poisoning patients using polymerase chain reaction restriction fragment length polymorphisms (PCR-RFLPs). The patient group consisted of 231 patients with DNS and 283 patients with no signs of lasting neurological damage (control population).

**Results:**

The frequency of the rs1784594 T allele was significantly lower in the DNS population (OR = 1.42, 95%CI: 1.08 − 1.87), as was the TT vs. CC genotype (OR = 1.95, 95%CI: 1.15 − 3.23) and the TT vs. CT + CC frequency (OR = 1.68, 95%CI: 1.32 − 2.49) compared to controls. Association analysis revealed a significant association between DNS and rs1784594 (*P* < 0.01) but not rs1893895 (*P* > 0.05). In female cases, the T allele frequency of rs1784594 was significantly lower in DNS patients compared to female controls (OR = 1.48, 95%CI: 1.01 − 2.17).

**Conclusion:**

These data suggest that the allelic variant of rs1784594 is a risk factor for DNS following acute CO poisoning, especially in females. The PARK2 protein may modulate the susceptibility to DNS, underscoring the importance of examining the relationship between other PARK2 polymorphisms and clinical outcome following CO poisoning.

## Background

Inhalation of carbon monoxide (CO) in quantities sufficient to cause systemic tissues hypoxia is termed acute CO poisoning. Carbon monoxide inhalation is a relatively common cause of home fatalities and a significant occupational hazard [1]. A major fraction of acute poisoning patients recover from the acute stage of CO intoxication only to exhibit a recurrence of neuropsychiatric symptoms after a latent period (usually 3 to 60 days) of normal or near normal neurological function termed the lucid interval. These symptoms are termed delayed neuropsychological sequelae (DNS) after CO poisoning [2,3] and are thought to arise from delayed post-hypoxic leukoencephalopathy (DPHL). The earliest epidemiological study of DPHL, taken from New York hospital records dating from 1925 to 1935, documented 13 cases of late-onset neurological and psychiatric symptoms following acute CO poisoning that meet current DNS diagnostic criteria [4]. The incidence of DPHL is higher in patients over 40 years of age and increases progressively with age, while it is rare in children under 10 years and shows no significant gender differences [5,6]. About 25% of DNS cases result in permanent neuropsychological deficits. Identification of the most vulnerable patient groups combined with early diagnosis may improve the quality of care and reduce permanent disability following CO toxicity [7].

The signs and symptoms of DNS, including gait and motor disturbances, cognitive impairments, anxiety, depression, and insomnia, resemble those of Parkinson’s disease (PD) and Parkinson-like syndromes [7,8], suggesting common sites of neural damage and similar pathogenic mechanisms. Mutations in the Parkinson protein 2 gene (PARK2) are associated with autosomal recessive juvenile Parkinson’s disease [9]. To study the risk of PD, several single-nucleotide polymorphisms (SNPs) of PARK2 have been investigated [10-12]. Given the clinical resemblance of PD and DNS, we examined if several PARK2 SNPs influence the susceptibility to DNS following CO poisoning.

In a previous study, we conducted pooling-based genome-wide association study in two independent samples of CO poisoning patients with or without DNS using the Infinium human 660 W-Quad array. Replicated associations were identified, and selected findings were confirmed by individual genotyping [13]. PARK2 was one of the promising genes (unpublished data). In the present case–control study, we compared the variant frequencies of two PARK2 gene polymorphisms, rs1784594 (C/T) and rs1893895 (G/A), in 514 Chinese-Han individuals from Northern Henan Province, to determine if these particular PARK2 variants influence DNS susceptibility.

## Methods

### Subjects

A total of 514 patients with acute CO poisoning were recruited from three affiliated hospitals of Xinxiang Medical University, Xinxiang 1st People’s Hospital and Xinxiang 2nd People’s Hospital, respectively, from October 2006 to October 2010. Demographic variables were shown in Table 1. Clinical diagnosis was made by specialty-trained neurologists. The DNS patients were diagnosed according to the following criteria [14,15]: (1) acute CO poisoning leading to coma in the previous 1 to 2 months, (2) an intervening “lucid interval” prior to the appearance of delayed symptoms, (3) delayed acute dementia indicating widespread cortical dysfunction as the main clinical manifestation, (4) EEG, CT, and/or MRI abnormalities, (5) the exclusion of other causes of dementia, and (6) older than 40 years. The acute CO poisoning patients (controls) were chosen by the following criteria: (1) a history of acute CO poisoning as evidenced by coma and elevated carboxyhemoglobin in blood at the time of presentation, (2) no signs of DNS 90 days or more following acute CO exposure, (3) exclusion of other etiologies, and (4) older than 40 years. This study was approved by our institutional Clinical Research Ethics Board and written informed consent was obtained from each patient involved in the study.

**Table 1:** Demographic variables of DNS (case) and control patients genotyped for the rs1784594 and rs1893895 polymorphism

**Characteristic**	**rs1784594**		**rs1893895**	
	**Cases(n = 180)**	**Controls (n = 262)**	**Cases (n = 217)**	**Controls (n = 226)**
Age	60.05 ± 9.83	56.24 ± 7.10	62.35 ± 8.31	58.05 ± 9.71
Sex (%)				
Male	94 (52.22)	119 (45.42)	120 (55.30)	107 (47.35)
Female	86(47.78)	143(54.58)	97(44.70)	119(52.65)
Educational level (%)				
Uneducated	52(28.89)	88(33.59)	65(29.95)	72(31.86)
Primary school	68(37.78)	99(37.79)	91(41.94)	74(32.74)
Middle school	60(33.33)	75(28.62)	71(28.11)	80(35.40)
Hypertension history (%)	67 (37.22)	119 (45.42)	162 (74.65)	154 (68.14)

Data presented as total number of instances (%) unless otherwise indicated.

### Genotyping

Genomic DNA was extracted from Peripheral blood samples from each subject using the RelaxGene Blood DNA System (Tiangen Biotech, Beijing, China). Five SNPs met association signal in pooling-based genome-wide association study (unpublished data, Additional file 1: Table S1). The biological functions and risks associated with individual SNP sites were evaluated using FASTSNP online service [16], and the GeneCards data base (http://www.genecards.org/). The SNPs ranked “high risk” and with minor allele frequency (MAF) ≥ 0.05 in the Chinese Beijing population in the HapMap database were chosen. Finally, the PARK2 gene polymorphisms rs1784594 and rs1893895 were chosen. The DNA fragment con-taining SNPs were amplified in reaction mixture con-taining Golden DNA polymerase (Gloden Fast PCR Kit, TIANGEN, Beijing, China) with primers 5′-GTTCACACCTTCTGCCTTGCTT-3′ and 5′- TCACAACACTGAGAGGCACTGG-3′ for rs1784594; 5′-GTGACAGGACCCAGCTGAAGAG-3′ and 5′-TGCAATGTCACAATCTTGGCTC-3′ for rs1893895. The conditions used for PCR amplification included an initial denaturation phase at 94°C for 2 min, followed by 35 cycles of 94°C for 15 s, 59°C for 5 s, and 72°C for 15 s, and a final extension phase of 72°C for 2 min. As a quality control, 100 randomly selected samples were genotyped by DNA sequencing.

PCR products were completely digested with the restriction enzyme (5U *Bsh1236I* for rs1784594, 5U *NlaIII* for rs1893895). The fragments were separated on 2% agarose gels and visualized under ultraviolet light after staining with ethidium bromide using the 100 bp DNA Ladder as a standard marker. The three genotypes resulting from digestion with *Bsh1236I* were AA (82 bp, 99 bp), AG (32 bp, 82 bp, 99 bp, 150 bp) and GG (99 bp, 150 bp) for rs1784594. Similarly, the three genotypes yielded by digestion with *NlaIII* were GG (122 bp, 223 bp), AG (122 bp, 223 bp, 345 bp) and AA (345 bp).

### Statistical analyses

All genetic analysis was performed using the SNPStats, a web tool [17]. Genotype and allele frequency were compared between two groups using the Pearson chi-square test with co-dominant model. Hardy–Weinberg equilibrium (HWE) was assessed using the chi-square test with one degree of freedom. Odds ratios (ORs) and 95% confidence intervals (95% CI) were calculated to evaluate the effects of alleles and genotypes. To evaluate interactions between SNP and sex, a global test for interaction was performed in the co-dominant model, in addition to a test for the interaction in the linear trend of the nested variable. Take into account a possible effect of age adjust their models by age. A two-tailed P-value ≤ 0.05 was considered significant.

## Results

We compared variants of the PARK2 gene SNPs rs1784594 (C/T) and rs1893895 (G/A) between CO poisoning patients presenting with delayed neurological sequelae (DNS, n = 231) and CO poisoning patients with no lasting neurological deficits (controls, n = 283). Of the total cases (514), 442 cases were genotyped for the rs1784594 SNP and 443 for the rs1893895, while a subset from each group (371) were genotyped for both. Mean age, sex ratio, and proportion of hypertensives were not significantly different between cases and controls genotyped for rs1784594 or rs1893895 (Age: *P* = 0.072 and *P* = 0.190; Sex Ratio: *P* = 0.053 and *P* = 0.114; Hypertensive status: *P* = 0.086 and *P* = 0.096) (Table 1), indicating well-matched groups. The SNPs were in Hardy-Weinberg equilibrium in both the case and control group (Table 2).

**Table 2:** Allele and genotype frequencies of the rs1784594 and rs1893895 polymorphisms in DNS cases and controls

**SNP**	**Genotype (%)**			**HWE( *P *)**	***χ***^**2**^		***P *value**	
					**Genotype**	**Allele**	**Genotype**	**Allele**
rs1784594	CC	CT	TT					
Cases (n = 180)	31 (17.2)	93 (51.7)	56 (31.1)	0.472	7.01	6.56	0.030	0.010
Controls (n = 262)	32 (12.2)	117 (44.7)	113 (43.1)	0.840				
rs1893895	GG	GA	AA					
Cases (n = 217)	74 (34.1)	93 (42.9)	50 (23.0)	0.051	0.36	0.36	0.836	0.550
Controls (n = 226)	81 (35.8)	98 (43.4)	47 (20.8)	0.090				

The genotype and allele frequencies of both polymorphisms are presented in Table 2. There was a significant difference in rs1784594 allele frequencies between DNS cases and controls (OR = 1.42; 95%CI: 1.08-1.87; *P* < 0.05). Furthermore, the 1784594 TT genotype frequency was significantly lower versus CC (OR = 1.95; 95%CI: 1.15-3.23; *P* < 0.05) and CT + CC (OR = 1.68; 95%CI: 1.11-2.54; *P* < 0.05) in DNS patients. When the model was adjusted by age, the significant remained (Table 3). In contrast, there were no significant associations between the rs1893895 G/A allelic variants and clinical outcome.

**Table 3:** Rs1784594 association with response Group (adjusted by age)

**Model**	**Genotype**	**Controls**	**Cases**	**OR (95% CI)**	***P *value**
Codominant	TT	113 (43.1%)	56 (31.1%)	1.00	0.03
	CT	117 (44.7%)	93 (51.7%)	1.62 (1.06-2.47)	
	CC	32 (12.2%)	31 (17.2%)	1.93 (1.07-3.47)	
Dominant	TT	113 (43.1%)	56 (31.1%)	1.00	0.0097
	CT-CC	149 (56.9%)	124 (68.9%)	1.69 (1.13-2.52)	
Recessive	TT-TC	230 (87.8%)	149 (82.8%)	1.00	0.16
	CC	32 (12.2%)	31 (17.2%)	1.47 (0.86-2.51)	
Overdominant	TT-CC	145 (55.3%)	87 (48.3%)	1.00	0.13
	CT	117 (44.7%)	93 (51.7%)	1.34 (0.92-1.97)	
Log-additive	---	---	---	1.44 (1.09-1.90)	0.011

To further examine the impact of rs1784594 on DNS risk, cases and controls were divided by gender. The female DNS cases exhibited a significant difference in allele frequencies (OR = 1.48; 95% CI: 1.01–2.17; *P* < 0.05) compared to female controls (Table 4). But there is no significant interaction between SNP and sex (Table 5).

**Table 4:** Genotype and allele frequencies of rs1784594 in female and male samples

** Sex**		**Genotype (%)**			***χ***^**2**^		***P *value**	
		**CC**	**CT**	**TT**	**Genotype**	**Allele**	**Genotype**	**Allele**
Male	Cases (n = 94)	16 (17.0)	48 (51.1)	30 (31.9)	3.101	2.622	0.212	0.105
	Controls (n = 119)	16 (13.4)	51 (42.9)	52 (43.7)				
Female	Cases (n = 86)	15 (17.4)	45 (52.3)	26 (30.2)	4.155	3.98	0.125	0.045
	Controls (n = 143)	16 (11.2)	66 (46.2)	61 (42.6)				

**Table 5:** Rs1784594 and sex cross-classification interaction table (n = 442, crude analysis)

**Genotype**	**Female**			**Male**			***P *value**		
	**Controls**	**Cases**	**OR (95% CI)**	**Controls**	**Cases**	**OR (95% CI)**	**Global test**	**Sex within SNP**	**SNP within sex**
TT	61	26	1.00	52	30	1.35 (0.71-2.57)	0.90	0.75	0.90
CT	66	45	1.60 (0.88-2.90)	51	48	2.21 (1.21-4.04)			
CC	16	15	2.20 (0.95-5.10)	16	16	2.35 (1.02-5.39)			

## Discussion

Mutations, deletions, and polymorphisms of the PARK2 gene have been associated with PD, cancer, susceptibility to bacterial infections, and other diseases [9,18-20]. Recently, a number of SNPs within PARK2 have been identified that are possible risk factors for PD [21-23]. Furthermore, early-onset PD, autism spectrum disorder, and progressive supranuclear palsy have also been associated with PARK2 mutations [24-26], suggesting that these PARK2 mutations disrupt neural development or decrease resistance to insult, leading to developmental deficits or increasing susceptibility to diseases such as DNS following CO poisoning.

In contrast to neurodegenerative diseases, most studies on DNS following CO poisoning have focused on clinical characteristics, and few studies have attempted to link the propensity for DNS with specific genotypes [7,27-29]. Our case–control study suggests that an allelic variant of PARK2, the rs1784594 polymorphism, might influence DNS risk in a Han population from Northern Henan, China.

We measured the genotype frequencies of two PARK2 polymorphisms, rs1784594 and rs1893895, and found that the C variant of rs1784594 may be a risk factor for DNS. The allele frequencies indicated that the TT genotype might lower the risk for DNS after CO poisoning by 1.7-fold compared to CC + CT. This suggested that the T allele of rs1784594 may confer resistance against DNS through some as yet unknown neuroprotective mechanism. There was also a significant association between C/T allele distribution and clinical outcome in females, but not with any specific genotype. No significant association was found between hypertension status and DNS risk. Even after controlling for hypertension, rs1893895 variant, and sex, the rs1784594 remained a significant risk factor for DNS following CO poisoning.

To determine the gender effect, genotype and allele frequency in both sexes were assessed. The female DNS cases exhibited a significant difference in allele frequencies of rs1784594 compared to female controls. But there is no significant interaction between SNP and sex. DNS was a disease resulting from interactions between environmental factors (CO) and an individual’s biological background. The aim of this study was to explore genetic susceptibility about the normal human’s different consequences after poisoning. The different caused by gender effect is hard to ignore, especially in endocrine, development, autoimmunity and stress reaction. Sex-specific association was often reported in other psychosis. The female-specific association may be attributed to the interaction between estrogen and genes related to brain development.

## Conclusions

In conclusion, we demonstrated a significant association between the PARK2 rs1784594 polymorphism and delayed neuropsychological sequelae following CO poisoning among the Han population from Northern Henan Province. Thus, allelic variants of the PARK2 gene may influence the susceptibility to DNS. It is unclear whether other SNPs within PARK2 also influence the susceptibility to DNS. Such studies may help reveal the functional mechanisms for DNS following CO poisoning and the role of PARK2 mutations in neurodegeneration.

## Abbreviations

DNS: Delayed neuropsychological sequelae; CO: Carbon monoxide; PD: Parkinson’s disease; PARK2: Parkinson protein 2; SNP: Single nucleotide polymorphism; DPHL: Delayed post-hypoxic leukoencephalopathy; EEG: Electroencephalography; CT: Computed tomography; MRI: Magnetic resonance imaging; PCR: Polymerase chain reaction.

## Competing interests

The authors declare that they have no competing interests.

## Authors’ contributions

Author RG and WL designed the study and wrote the protocol. FL prepared the the first draft of the manuscript. Author FL and YZ finished the biological experiments. Author JG managed the literature searches and analyses. Author WL and XW undertook the statistical analysis. Author XW, HZ, and PZ collected clinical data. All authors contributed to and have approved the final manuscript.

## Pre-publication history

The pre-publication history for this paper can be accessed here:

http://www.biomedcentral.com/1471-2350/14/99/prepub

## Supplementary Material

Additional file 1: Table S1 SNPs of PARK2 met association signal in pooling-based genome-wide association study. Click here for file
