# Supplementary material for: A PARK2 polymorphism associated with delayed neuropsychological sequelae after carbon monoxide poisoning
Source: BMC Med Genet. 2013 Sep 25;14:99. doi: 10.1186/1471-2350-14-99 (PMC3849006; doi:10.1186/1471-2350-14-99)
Supplement: Additional file 1: Table S1 — SNPs of PARK2 met association signal in pooling-based genome-wide association study. [file 1471-2350-14-99-S1.doc]

**Table S1** SNPs of PARK2 met association signal in pooling-based genome-wide association study

| SNP | Position | *P* value | |
| --- | --- | --- | --- |
|  |  | Female | Male |
| rs1784597 | 162295899 | 8.41×10^-7^ | 2.97×10^-6^ |
| rs1624390 | 162296972 | 3.48×10^-7^ | 2.22×10^-6^ |
| rs1784594 | 162302724 | 1.84×10^-6^ | 5.78×10^-6^ |
| rs1893895 | 162360677 | 6.35×10^-5^ | 7.68×10^-6^ |
| rs9364635 | 162362566 | 5.37×10^-5^ | 4.33×10^-5^ |
